# Short-Chain Fatty-Acid-Producing Bacteria: Key Components of the Human Gut Microbiota

**DOI:** 10.3390/nu15092211

**Published:** 2023-05-06

**Authors:** William Fusco, Manuel Bernabeu Lorenzo, Marco Cintoni, Serena Porcari, Emanuele Rinninella, Francesco Kaitsas, Elena Lener, Maria Cristina Mele, Antonio Gasbarrini, Maria Carmen Collado, Giovanni Cammarota, Gianluca Ianiro

**Affiliations:** 1 Department of Medical and Surgical Sciences, Digestive Disease Center, Universitary Policlinic Agostino Gemelli Foundation IRCCS, 00168 Rome, Italy; william7134@gmail.com (W.F.); porcariserena89@gmail.com (S.P.); francesco.kaitsas01@icatt.it (F.K.); elena.lener01@icatt.it (E.L.); antonio.gasbarrini@unicatt.it (A.G.); giovanni.cammarota@unicatt.it (G.C.); 2 Department of Translational Medicine and Surgery, Catholic University of the Sacred Heart, 00168 Rome, Italy; marco.cintoni@unicatt.it (M.C.); emanuele.rinninella@unicatt.it (E.R.); mariacristina.mele@policlinicogemelli.it (M.C.M.); 3 Institute of Agrochemistry and Food Technology-National Research Council (IATA-CSIC), 46022 Valencia, Spain; mbernabeu@iata.csic.es (M.B.L.); mcolam@iata.csic.es (M.C.C.); 4 Clinical Nutrition Unit, Department of Medical and Surgical Sciences, Universitary Policlinic Agostino Gemelli Foundation IRCCS, 00168 Rome, Italy

**Keywords:** gut microbiota, short-chain fatty acids, SCFA-producing bacteria, gut health, prebiotics, probiotics, diet

## Abstract

Short-chain fatty acids (SCFAs) play a key role in health and disease, as they regulate gut homeostasis and their deficiency is involved in the pathogenesis of several disorders, including inflammatory bowel diseases, colorectal cancer, and cardiometabolic disorders. SCFAs are metabolites of specific bacterial taxa of the human gut microbiota, and their production is influenced by specific foods or food supplements, mainly prebiotics, by the direct fostering of these taxa. This Review provides an overview of SCFAs’ roles and functions, and of SCFA-producing bacteria, from their microbiological characteristics and taxonomy to the biochemical process that lead to the release of SCFAs. Moreover, we will describe the potential therapeutic approaches to boost the levels of SCFAs in the human gut and treat different related diseases.

## 1. Introduction on Human Gut Microbiota

The human gut microbiota comprises an ecological community that includes bacteria, yeasts, viruses and parasites, yielding nearly 100 trillion microorganisms [1,2,3,4]. At birth, the human gut is almost sterile, and is soon populated by the mother microbiome. The type of delivery, vaginal or cesarean, plays a main role in the composition of the gut microbiome in the newborn [4]. Healthy gut microbiota mainly comprises (nearly 90%) two phyla, Firmicutes and Bacteroidetes, and also contain less-represented phyla, such as Proteobacteria, Verrucomicrobia, or Actinobacteria [5]. The phylum Firmicutes includes several genera, of which the most common (up to 95% of the total) are *Lactobacillus*, *Bacillus*, *Enterococcus*, *Ruminicoccus* and *Clostridium* [5]. the *Bifidobacterium* genus is the most abundant of the Actinobacteria phyla; however, the phylum itself is less present overall [5]. Many of the microorganisms in the normal microbiota are opportunistic pathogens [6,7]. The distinction between opportunistic pathogens and commensal microbes is largely determined by the host immunity, rather than any intrinsic feature of that microorganism. Therefore, competent human immunity can shape potential pathogenic microbes into symbiotic ones. Microbes also colonize other sites of the gastrointestinal tract, and the composition of the esophageal or gastric microbiome differs from that of the microbiome in the gut [8,9].

Gut microbiota is one of the key components of the intestinal ecosystem, and plays an essential role in human health, including a barrier effect against pathogens, in the shaping and maturation of immunity, in the regulation of metabolic intake and in the absorption of nutrients and drugs [10,11,12,13,14,15,16]. The imbalance of gut microbiota has been associated with several gastrointestinal [17] and extraintestinal disorders [18,19], and several therapeutic approaches, including diet [20], antibiotics [21], prebiotics, probiotics or postbiotics [22,23] or fecal microbiota transplantation [24], are increasingly investigated for use in microbiome-based disorders. One of the most relevant therapeutic pathways of microbiome modulation includes the restoration of the levels of short-chain fatty acids (SCFAs), microbial metabolites that are essential for human health. In this review article, we will provide an overview of the functions of SCFAs in human health, SCFA-producing bacteria, and potential ways to boost their levels in the human gut.

## 2. Structure and Functions of SCFAs in Physiological Conditions

Short-chain fatty acids (SCFAs) are organic acids whose carbon chain is composed of less than six carbons. Among these, acetate (C2), propionate (C3) and butyrate (C4) are the most represented [25]. Acetate contributes to approximately 60% of the total SCFAs while propionate and butyrate comprise 20% each [26]. Additional acids, including lactate isomers, valerate, and branched chain SCFAs such as isobutyrate and iso-valerate, can be found in our gut metabolome (the metabolites of our gut microbiome), but their levels are noticeably lower compared with the main acids [27].

The main functions of SCFAs are carried out with the aid of Free Fatty Acid Receptor 2 (FFAR2) and FFAR3, while FFAR1 and FFAR4 are used by medium- and long-chain fatty acids. FFARs are G-protein-coupled transmembrane receptors located on the surface of many different cells (neurons, colonocytes, pancreatic cells, neutrophils, adipocytes, enteroendocrine cells, etc.) [28]. Acetate, a C2 SCFA, is more effective in the activation of the FFAR2 receptor, while propionate, a C3 SCFA, mainly effects the FFAR3 receptor. These receptors play key roles in various cells. FFAR2 and FFAR3 could mediate both the anti-inflammatory effect of acetate and propionate, and the proinflammatory effect of butyrate on innate immune system cells [29]. Moreover, the action of those two receptors may influence the energy consumption of neurons [30], insulin secretion from Langerhans islets beta cells [31,32] and enteroendocrine function [33,34].

The effects of SCFAs on the human gut are mediated by the presence of SCFA transporters on colonic epithelium. These transporters can be grouped into three main transporter classes: proton-coupled transporters, such as MCT1 and MCT4; sodium-coupled transporters, using the energy of two sodium ions, such as SMCT1; and ATP-dependent transporters, such as ABCG2, also known as breast cancer resistance protein (BCRP) [35].

SCFAs have several beneficial effects on human health, at different levels and on body sites.

First, SCFAs promote the integrity and permeability of the gut barrier in different ways. These molecules, mainly butyrate, increase the concentration of tight junctions, such as claudin-1, zonula occludens-1 and occludin through the upregulation of genes that encode for these proteins [36]. Moreover, butyrate is able to strengthen the mucus layer of the gut epithelium by increasing the expression of Mucin 2 [37]. Butyrate is also involved in the modulation of oxidative stress, as it reduces H_2_O_2_-induced DNA damage, restoring the levels of antioxidant glutathione. Additionally, SCFAs can induce both the differentiation and apoptosis of colonic cells, ideally preventing the development of colon cancer, as discussed further in this Review [38].

SCFAs also play an important role in the regulation of several physiological pathways within the nervous system. First, SCFAs modulate brain-induced intestinal gluconeogenesis. In particular, when propionate is absorbed and passes through the portal vein, it activates the FFAR3s present on the surface of afferent periportal neurons [39]. SCFAs also regulate the inhibition of histone deacetylase (HDAC), with a potential impact on several neuropsychiatric diseases such as depression, schizophrenia and Alzheimer’s disease [40]. Moreover, SCFAs control systemic and neuroinflammation through the modulation of functions and structures of microglia cells, resulting in the modulation of emotion, cognition and mental disorders. Additionally, high concentrations of SCFAs seem to be related to the major expression of neurotrophic factors [41]; SCFAs may induce the expression of tryptophan 5-hydroxylase 1, an enzyme involved in serotonin biosynthesis [42], and there is also evidence that they may positively affect the brain barrier’s integrity [43,44].

SCFAs, especially acetate, are also involved in the regulation of appetite and human metabolism. In animal models, diets with a high abundance of fermentable carbohydrates, whose catabolism in the colon generates SCFAs, relate to a minor appetite [45]. Moreover, acetate may reduce body weight through the secretion of glucagon-like peptide 1 and peptide YY [46]. SCFAs are also able to modulate both glucose and lipid metabolism. Propionate suppresses hepatic gluconeogenesis [47], while both acetate and butyrate reduce lipogenesis and increase leptin secretion [48,49,50,51]. Furthermore, SCFA administration in animal models seems to reduce liver steatosis [52,53], and vinegar, a food rich in acetate, was demonstrated to be useful in reducing body weight, serum triglycerides and body fat mass [54]. However, most experiences on humans are biased by a small sample size, and more evidence from adequately sized clinical studies is needed to understand the effects of SCFAs on lipidic metabolism [55].

Increasing evidence suggests that SCFAs are able to influence other components of cardiometabolic health. Increased levels of butyrate and propionate are associated with the reduction in blood pressure [56] and plasminogen activator inhibitor-1 (PAI-1) levels, a pro-thrombotic factor [57].

Notably, SCFAs have a relevant impact on both innate and adaptive immunity. Regarding innate immunity, SCFAs can act directly on neutrophils, reducing their production of reactive oxygen species (ROS) and myeloperoxidase (MPO), and can even enhance their apoptosis [58]. They also reduce the chemotaxis of inflammatory cells due to a decrease in the expression of monocyte chemoattractant protein-1 (MCP-1), vascular cell adhesion molecule-1 (VCAM1) and chemokines signals [59,60]. In addition, regarding the T cell lineage, SCFAs can increase the T_reg_ cell number and their activity and inhibit CD4+ [61,62]. Finally, treatment with SCFAs, and especially with butyrate, is able to reduce gut inflammation, reducing the NF-κB signaling pathway and enhancing the expression of anti-inflammatory cytokines such as IL-10 [63].

## 3. SCFAs-Producing Bacteria of the Human Gut Microbiota

Intestinal microbiota is the main source of bacteria, producing SCFAs through the degradation of substrates, mainly non-digestible polysaccharides, including dietary fibers and resistant starches. Interestingly, the concentration of SCFA fluctuates throughout our life, and these longitudinal changes appear to be related to the composition of our gut microbiome, which also varies during our life cycle [64,65]. Notably, the variety of our diet, which changes during our life, has a heavy influence on the quantity of SCFAs released in the intestine, modulating the amount of substrate sources for SCFAs-producing bacteria [66].

**Figure 1:**
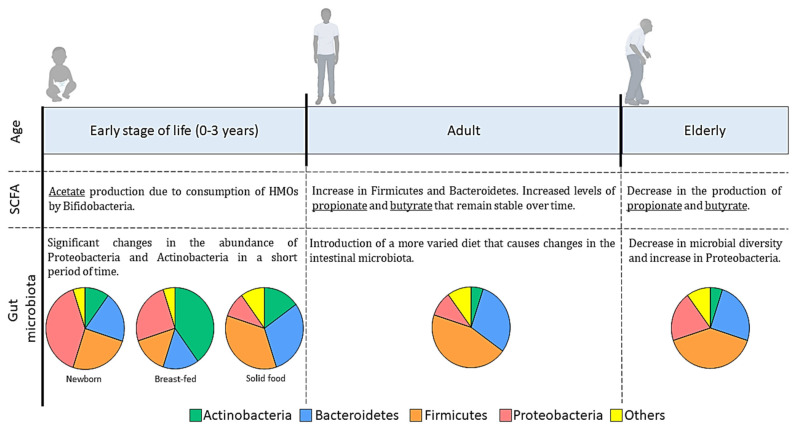
Microbiota development and short-chain fatty acid production at different stages of human life [65,67,68,71]. Manuel Bernabeu Lorenzo, Maria Carmen Collado. Underline: emphasis.

Specifically, in early life, the microbiota evolves from being dominated by *Enterobacteriaceae* to being dominated by *Bifidobacteriaceae* while later, along with the end of breastfeeding, an increase in the abundance of Firmicutes can be observed [67,68]. Firmicutes species, including *Lactobacillaceae*, *Ruminococcaceae* and *Lachnospiraceae*, are able to break down complex polysaccharides and other sugars through hydrolysis, resulting in the production of butyrate and other SCFAs [69,70]. Finally, in older age, the microbiota changes again and the abundance of *Enterobacteriaceae* increases [68,71]. These changes in the microbiota are also reflected in the production and diversity of SCFA, or even in the profile of branched short-chain fatty acids (BCFA) [72,73], as shown in Figure 1.

The levels of the SCFAs detected in the gut, acetate, propionate and butyrate are affected by these age-driven changes in the human gut microbiota. Acetate concentration is higher in the early stages of life as it is the main product of *Bifidobacteria* strains that characterise the infant gut microbiota: strains *B. bifidum*, *B. infantis* and *B. breve* are the main players [68,74] *Bifidobacterium* strains are able to utilize the human milk oligosaccharides (HMOs) [75] to obtain carbon and energy [76]. HMOs have galactose and glucose as their main components and *Bifidobacteriaceae* can transform galactose into glucose. The consumption of this glucose is associated with an increase in acetate and formate, 1,2-propanediol, and lactate, as has been seen by the increase in such metabolites during in vitro Bifidobacterium co-cultivation [77].

Healthy infant microbiota development is characterized by the predominance of certain *Bifidobacterium* species, and the absence of HMO-metabolizing bifidobacterial genes has been correlated with systemic and intestinal inflammation [78,79]. In addition, in vitro studies suggest that acetate-producing *Bifidobacterium* is able to protect against bacterial infections, as has been observed with pathogenic *E. coli* [80]. Interestingly, the produced acetate benefits the growth of propionate and butyrate-producing bacteria and, at the same time, butyrate favors the growth of *Bifidobacterium*, leading to a cross-feeding between SCFA-producing bacteria [72,81].

Acetate production requires substrates described as acetogenic fibers (inulin, galacto-ligosaccharides, etc.) [66]. Those fibers may then enter two possible pathways: acetogenesis or carbon fixation pathway. Acetogenesis is the production of acetate, mediated by homoacetogenic bacteria, which can use both H_2_ and CO_2_, while the carbon-fixation pathway produces acetate directly from CO_2_ [66,82,83].

Gut propionate levels increase after the cessation of breastfeeding and the introduction of a more varied diet. Due to the presence of more diverse food, the microbiota change, with a greater proportion of Firmicutes, mainly of the Clostridia class [73,84]. Propionate can be formed from the fermentation of sugars in three ways. The succinate pathway processes hexoses and pentoses (via a pathway that, thanks to vitamin B12, converts succinyl-Coa into proprionate), while the acrylate pathway converts lactate into propionate, and, through the propanediol pathway, deoxy sugars (e.g., fucose and rhamnose) are processed [85]. Bacteria that use the succinate route belong mostly to the Bacteroidetes (*Prevotella* spp.) and Negativicutes classes, such as *Phascolarctobacterium succinatutens* or *Veillonella* spp. [86,87,88]. In the case of the acrylate route, the best-studied bacteria are *Coprococcus* spp. which belongs to the *Lachnospiraceae* family. Strikingly, some of the members of this genus are capable of producing butyrate in addition to propionate, depending on the initial substrate used [85]. Finally, propanediol-dependent metabolic pathways have been observed in *Roseburia inilivorans* and in *Blautia* species, which also belong to the *Lachnospiraceae* family [89,90].

Among the three main SCFAs, butyrate has the greatest impact at the physiological level; therefore, the bacteria that produce this have the highest relevance.

Butyrate derives from the condensation of two molecules of acetyl-CoA to form acetoacetyl-CoA, which is then gradually reduced to butyryl-CoA. Butyryl-CoA is then transformed in butyrate via butyryl-CoA:acetate CoA-transferase or via phosphotransbutyrylase and butyrate kinase [89].

Butyrate-producing microbial communities in the gut are essential for maintaining a healthy gut environment. These communities play a critical role in limiting the entry and establishment of other bacteria, particularly harmful pathogens. The production of butyrate by these bacteria is necessary for the colonocytes to generate energy and to increase epithelial oxygen consumption [91,92]. This, in turn, helps to maintain an anaerobic gut environment that is harsh for opportunistic aerobic pathogens such as *Salmonella* and *E. coli* [93,94]. As previously mentioned, the main species involved in the production of butyrate are found within the *Lachnospiraceae* and *Ruminococcaceae* families [95,96]. As an example of the *Lachnospiraceae* family, the genera *Roseburia* and *Blautia* have been seen as being related to the maturation of the immune system or intestinal inflammation [97,98]; *E. hallii*, which interacts with other SCFA-producing bacteria, provides another example [99]. *Roseburia intestinalis* and acetogenic species work together in a cooperative manner to carry out butyric metabolism, which occurs without the production of H_2_ [100]. On the other hand, when degrading L-fucose and fucosyllactoses, *E. hallii* engages in a trophic interaction with *B. breve* and *B. infantis*, highlighting the metabolic versatility of *E. hallii,* as it can utilize the intermediates produced during bifidobacterial oligosaccharide fermentation [101]. In the case of the *Ruminococcaceae* family, *Faecalibacterium prausnitzii* represents up to 5% of the fecal microbiota of healthy adults, being one of the most abundant butyrate-producing bacteria [102,103]. Different studies indicate the relationship between lower levels of this bacterium and various diseases, such as inflammatory bowel disorders (IBD) [104,105]. While most butyrate-producing microbes are classified under the Firmicutes phylum, research has indicated that some species from Actinobacteria, Fusobacteria or even Proteobacteria can also generate butyrate [106,107].

This finding adds to the evidence that, beyond carbohydrates, SCFAs can also derive from the fermentation of proteins and amino acids. Rasmussen et al. showed that the addition of albumin resulted in an increase in fecal C2-C5-SCFAs and that the incubation of fecal samples with specific amino acids resulted in the increase in specific SCFAs: hydroxyproline, serine, and glutamate resulted in an increased concentration of both acetate and butyrate, whereas histidine resulted in the increased production of acetate, and propionate was also produced from aspartate [108]. These findings were confirmed by Macfarlane et al., who found an increase in the concentration of SCFAs after the application of casein and bovine serum albumin to washed-gut bacteria, obtained from fresh faeces [109].

Although the main SCFAs are acetate, propionate and butyrate, other minor ones such as formate and lactate are also produced as a result of microbiota metabolism. In this way, the producing bacteria of these SCFAs and their role in the physiology of the host are drawing attention.

Formate appears as one of the end products of *Bifidobacteria* metabolism consuming HMOs, as occurs with acetate, lactate and 1,2-propanediol [77]. The formate can derive from an inadequate metabolism of LnNT, one of the main HMOs, by *B. infantis*, which leads to the production of formate and ethanol, instead of acetate and lactate [110]. *E. hallii* has also been identified as a format-producing bacteria. In this case, the production is carried out from 1,2-propanediol, obtaining both formate and propionate and butyrate [101,111]. This example further shows the relationship between SCFA-producing bacteria and their metabolisms.

Besides, as previously indicated, lactate is one of the metabolites produced in the early stages of life, along with formate and acetate. Therefore, lactate-producing bacteria are related to the first stage of the intestinal microbiota, mainly *Bifidobacteria* and *Lactobacilli*, but also *Staphylococci* and *Streptococci* [112,113]. Lactate can be used by the bacteria present in gut microbiota, such as *E. hallii* and *Anaerostipes caccae*, which produce propionate and butyrate as a result [85,86]. Although the main SCFA-producing bacteria have been described, further research is needed in this field. The development of new methodologies and the use of novel approaches, mainly whole-genome sequencing and metabolomics, will allow for the identification of specific SCFAs-producing bacteria and also increase knowledge of the interactions between them and our organism, and the related consequences for human health.

## 4. SCFAs Production as a Marker of Healthy Gut Ecosystem

SCFAs production is essentially a biochemical process carried out by gut bacteria, but it may reflect the homeostasis of the gut. Several lines of evidence support the concept of SCFA production as a marker of a healthy gut ecosystem.

First, SCFAs-producing taxa are usually commensal, beneficial bacteria, with a direct positive effect on the gut barrier and immunity beyond the production of SCFAs [55,114].

Moreover, the mucus colonic layer is another element that is positively influenced by SCFAs.

The health of the mucus barrier can be considered a result of host–microbiota and within-taxa microbiome cross-talk, and these interactions also influence the production of SCFAs. As an example, the phenomenon of cross-feeding allows for taxa such as *Akkermansia muciniphila*, which is able to use the carbohydrates included in the mucus as a source of energy, to release oligosaccharides and acetate in the intestinal microenvironment and feed other bacteria [115,116]. Those molecules are then captured by bacteria such as *Eubacterium hallii*, which then can produce propionate, butyrate and vitamin B_12_, which are released in the lumen and can exert their trophic effects on colonocytes [111,117].

Conversely, SCFAs, mainly butyrate, may influence both the quality and the quantity of the mucus produced by the intestinal goblet cells. The induction of mucus production by SCFAs can be assessed by measuring the expression of MUC2, a gene coding for mucin 2. In mice, butyrate enemas may enhance MUC2 expression in the colon, indicating an increase in its production, and can reduce mucus’ thickness [118]. Butyrate may enrich the mucus layer, improving the processes of sulphation, acetylation, and sialylation, and creating different kinds of mucins that can be used as substrates for different metabolic pathways by the gut bacteria [119]. Sialylation plays a key role in mucus homeostasis, and its defects are linked to inflammatory diseases [120]. Moreover, butyrate favors the adherence of Bifidobacteria to the epithelial barrier, reducing the adhesion of potentially pathogenic species such as *E. coli* [121].

This hypothesis is strengthened by the evidence of SCFA imbalances in disease conditions.

As an example, in ulcerative colitis (UC), both detrimental shifts in the gut microbiome (and, more specifically, a reduction in SCFA-producing bacteria) and alterations in the mucus layer can be found [122]. Although the pathogenesis of UC and other chronic conditions is still poorly known, the complex relationship between the gut microbiome, SCFAs, colonocytes and intestinal mucus appears to be a promising therapeutic target, and deserves further research [104,123].

## 5. Mechanistic Involvement of SCFAs in the Development of Human Diseases

SCFAs play a significant anti-inflammatory role in the regulation of immune function [58], taking part in the prevention of various inflammatory chronic disorders [104,124,125,126].

In gut diseases, both acute and chronic inflammation are relevant. Transient acute inflammation, an essential defense mechanism of the immune system against injurious stimuli, is of particular relevant [127,128]. In this condition, when cells are damaged, instead of directly targeting the injurious stimuli, such as any invading viruses or bacteria, the immune system will use the “self-destroy and rebuild” strategy, targeting the damaged cells. By using a programmed cell death such as pyroptosis [129] and necroptosis [130] to actively destroy the cells, stimuli such as viruses or bacteria are also effectively cleared. On the other hand, chronic inflammation develops when the stimulus cannot be removed and is associated with diseases like IBDs, where SCFAs play a key role [131].

### 5.1. SCFAs and IBD

IBD includes chronic inflammatory disorders of the gastrointestinal tract associated with a gut microbiota imbalance. Patients with IBD are known to share, compared with healthy subjects, a reduction in butyrate producers of the Firmicutes phylum, mainly Roseburia spp and *Faecalibacterium prausnitzii*, and an increase in opportunistic bacteria [104,132].

In addition to a reduced SCFAs production, the uptake and oxidation of butyrate appears to be inhibited in patients with UC [133]. This leads to a weakening of their anti-inflammatory activity, thus promoting disease progression. More specifically, propionate and butyrate stimulate T-reg proliferation and function through GPR-43 pathways and HDACs’ inhibition [134,135,136]. SCFAs also lead to a downregulation of proinflammatory cytokines levels because of the inhibition of NF-κB and HDCAs activity [137,138,139], and to an increase in the anti-inflammatory ones through GPCRs [60].

Furthermore, acetate controls tissue homeostasis through NLRP-3 activation [140] and butyrate regulates the intestinal barrier, which is known to be impaired in IBD, through increased AREG, IL-22 and claudin-1 production [141,142].

### 5.2. SCFAs and Colorectal Cancer (CRC)

CRC is a multifactorial disease and the gut microbiota play an important role in its development [143]. Patients with CRC showed an increase in pathogenic bacteria (e.g., *Fusobacterium nucleatum*) and a depletion in butyrate producers [138,144,145]. The reduced production of SCFAs leads to a pro-inflammatory environment, which can contribute to the initiation and progression of CRC [146]. In addition, butyrate can change redox state and D-glucose metabolism, enhancing cancer cells’ apoptosis [147], while the inhibition of HDCAs regulates the expression of p21, arresting cell cycle and consequent cancer proliferation [148]. Proliferation is also inhibited by propionate via GPR-43, which is often lost in colon cancer cells [124].

### 5.3. SCFAs and Cardiovascular Diseases (CVDs)

There is a large body of evidence suggesting that SCFAs play a role in the pathogenesis of CVDs, a group of disorders that include hypertension and atherosclerosis. A reduction in butyrate producers in the gut microbiota and the deficient intestinal absorption of SCFAs have been observed in patients with hypertension [149,150]. Moreover, SCFAs appear to have a dual effect on the regulation of blood pressure. For example, when binding Olfr-78, acetate and propionate lead to renin release, increasing blood pressure [151]. By contrast, when binding GPR-41, they reduce blood pressure via vasodilatation [152], which is also obtained by the effect of butyrate on afferent vagal terminals [153]. In atherosclerosis, a similar pathway has been noted [154], as SCFAs, mainly butyrate, appear to play a protective role in the regulation of inflammation and stabilization of plaques by downregulating the expression of CCL-2, VCAM-1, and MMP-2, resulting in the lower migration of macrophages, increased collagen deposition and ultimate plaque stability [155].

### 5.4. SCFAs and Metabolic Diseases

As anticipated above, SCFAs regulate metabolic pathways and food intake, thereby playing a role in the development of metabolic diseases. Obesity is associated with an imbalance in the gut microbiota, mainly an increased Firmicutes/Bacteroidetes ratio, and an increase in fecal-SCFAs [126,156], although circulating SCFAs are reduced [157]. Type 2 diabetes (T2D) is instead characterized by a decrease in butyrate producers in the gut microbiota [158].

Normally, SCFAs moderate food intake, stimulating the secretion of satiety hormones such as PYY and GLP-1 via GPR-41 and GPR-43 [159,160] and through the inhibition of HDACs [161]. Furthermore, acetate can cross the blood–brain barrier, causing a decreasee in appetite [45]. SCFAs can also improve glucose homeostasis in an AMPK-dependent manner involving PPARγ-regulated effects on gluconeogenesis and lipogenesis [48]. Moreover, propionate enhances glucose-stimulated insulin release via GPR-43 and increases β-cell mass [162]. SCFAs can stimulate adipocyte differentiation [163,164] and decrease lipid plasma levels through the inhibition of lipolysis and stimulation of lipogenesis [165,166,167] and cholesterol plasma levels, enhancing its hepatic uptake [168].

Overall, these mechanistic pathways of SCFAs in different disorders pave the way for the therapeutic use of SCFAs in clinical practice. Table 1.

## 6. Therapeutic use of SCFAs in Clinical Practice

Considering the involvement of SCFAs in the colon physiopathology, and also considering the effects those molecules have at the cardiovascular and metabolic levels, many studies have investigated the potential of SCFAs as a therapeutic option for both intestinal and cardiometabolic disorders.

Several lines of evidence for the use of SCFAs in intestinal disorders derive from mouse models. Butyrate enemas were effective in improving symptoms, inflammation and the sodium absorption of colonocytes in mice with experimental distal colitis [169]. However, these pre-clinical findings were not replicated in humans, regardless of the disease.

In a randomized placebo-controlled trial of 91 patients, where enemas of acetate, propionate and butyrate were used to treat left-sided UC, SCFAs were not more effective than placebo in improving the clinical picture, only levels of mucin depletion before and after treatment [170].

In a recent systematic review of randomized controlled trials using butyrate enemas in IBD, the study concluded that, for butyrate, enemas are not effective in UC, while for Crohn’s disease, more studies are needed to clarify the role of SCFAs [171].

Oral butyrate supplementation was also valued for IBDs. In UC patients, sodium butyrate microcapsules were effective in reducing the Mayo score and faecal calprotectin levels compared to mesalamine alone [172]. Thus, in a bigger, randomized, placebo-controlled study conducted on paediatric patients, there was no significative difference between the standard-of-care therapy and the addition of sodium butyrate oral capsules in both newly diagnosed Crohn and UC [173].

In a systematic review of SCFAs enemas treatment for late-radiation proctitis, patients experienced a significant decrease in the weekly episodes of rectal bleeding and consequently higher levels of haemoglobin in only one study, without relevant results in other studies [174,175].

For IBS, data from in mouse studies show conflicting results about the role of SCFAs in the pathogenesis and physiopathology of this condition and its influence on visceral hypersensitivity [176,177,178]. Nevertheless, in human studies, a reduction in SCFA-producing bacteria was demonstrated for IBS-D and IBS-M [179], and the administration of butyrate in a triglyceride matrix was significantly effective in reducing the clinical severity of this disease [180]. However, double-blind, randomized, placebo-controlled studies are necessary to better evaluate the impact of this supplementation for IBS.

A prospective placebo-controlled randomized study demonstrated a significant impact of butyrate microcapsules in reducing episodes of diverticulitis in patients with diverticular disease [181]. These promising but preliminary findings support further research in this field.

Several pre-clinical lines of evidence that support the role of SCFAs in cardiometabolic disorders have recently been revealed. In mice fed a high-fat diet, the addition of butyrate was associated with a reduction in hepatic lipogenesis and hepatic steatosis, as well as with improvements in hepatic function and lipid profile, suggesting a possible role for SCFA supplementation in NAFLD [182].

In another mouse model, butyrate reduced heart ischemia-reperfusion damage [183], improved vascular function and reduced tension [184,185,186]. However, as in gastrointestinal disorders, these promising pre-clinical findings were not replicated in human studies [187]. Oral therapy with butyrate was not effective in improving metabolic outcomes in patients with diabetes [188] or with metabolic syndrome [189].

One reason for these unsatisfactory results is the therapeutic formulation of SCFAs, as butyrate supplements cannot reach high concentrations in the gut when administered by mouth. Recently, the colonic-delivery formulation of butyrate was found to positively affect quality of life and the gut microbiota composition of patients with UC [190], suggesting that this approach deserves further research.

## 7. How to Foster the Production of SCFAs in the Human Gastrointestinal Tract

**Figure 2:**
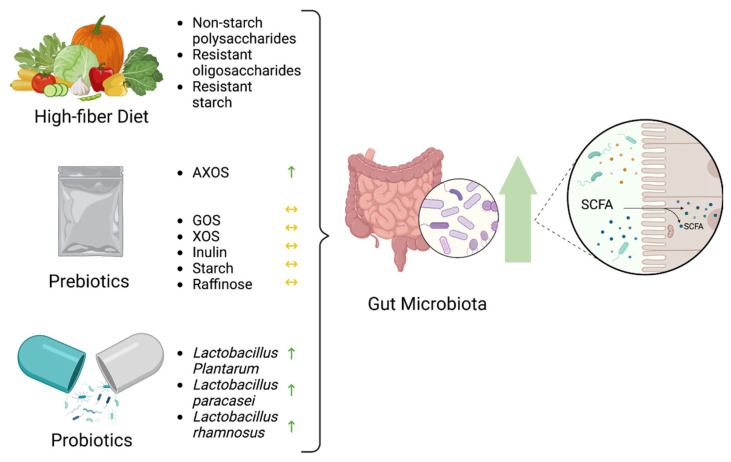
Established strategies that can be used in clinical practice to boost the microbiome-dependent production of short-chain fatty acids. Green arrow: increases the levels of SCFAs; yellow arrow: doesn’t significantly change the level of SCFAs. Marco Cintoni, Emanuele Rinninella.

Diet can influence and modulate the gut microbiota of individuals [191]. The major constituents of a normal diet, called macronutrients, are carbohydrates, protein, and fats. Fibers, defined as nondigestible carbohydrates composed of more than three monomeric units, could be considered the “fourth macronutrient”; fermentable fibers are used as substrates by colonic microbes, from which they are transformed into various metabolites including SCFAs [192]. Dietary fiber, prebiotic fiber supplements, and probiotics can modulate the gut microbiota and increase the overall production of SCFAs, as shown in Figure 2.

### 7.1. Diet

Even if there is not high homogeneity between studies on this issue, there is a general agreement that diets rich in fiber increase the amount of SCFA, in particular, acetate and butyrate [193]. Several studies reported that dietary interventions could increase the SCFA-producing bacteria. Studies focusing on the effects of high-fiber diets, such as Mediterranean, vegetarian, and vegan diets, on patients affected by IBD, resulted in improvements in microbiome outcomes (increase in alpha diversity, increase in specific microbial population, etc.), in laboratory exams (reduction in C-reactive protein (CRP), reduction in fecal calprotectin, etc.), and IBD-specific outcomes (i.e., reduction in Mayo Score), as well as in an increase in SCFA levels [194]. Likewise, a high-fiber diet led to a significant reduction in glycated hemoglobin, and an increase in glucagon-like peptide-1 (GLP-1) production, Bifidobacteria count, and total SCFA amount in patients affected by T2D [195]. On the other hand, studies evaluating the use of ketogenic diet (a low-carbohydrate, high-fat diet able to induce physiological ketosis) showed a decrease in beneficial bacteria (i.e., *Bifidobacteria*, *Eubacterium rectale*, *Roseburia*) and total bacterial count and abundance [20]. As a consequence, a ketogenic diet may induce a reduction in both total SCFAs and their single components [196].

### 7.2. Prebiotics

Prebiotics, defined as «substrates that are selectively utilized by host microorganisms conferring a health benefit», are widely used to increase SCFA levels [197]. Some studies showed an increase in total SCFAs, butyrate, acetate, and propionate, using arabinoxylan oligosaccharides (AXOS) at high doses (above 7.5 g per day) [198], and similar results were obtained if AXOS was administered through fiber-enriched food [199,200]. Studies performed on healthy people using different prebiotics, such as xylooligosaccharides, inulin, resistant starch, raffinose, and galactooligosaccharides, did not show any impact regarding SCFA levels [201]. In patients with T2D, resistant starch seems to increase SCFA levels, leading to a reduction in intestinal permeability, inflammation, and circulating cytokines [202]. The increase in SCFAs was also observed in patients during enteral nutrition treated with fiber-enriched formula, with a general reduction in diarrheal events [203,204].

### 7.3. Probiotics

Since the modulation of gut microbiota is a key way to induce a boost in SCFA levels, the administration of probiotics could be the most effective strategy. Both in vitro and in vivo studies confirmed a positive role of probiotics in increasing SCFAs [205]. The most used probiotics are those of *Lactobacillus* genera, mainly *Lactobacillus plantarum* [206,207], *Lactobacillus paracasei* [207], and *Lactobacillus rhamnosus* [208]; all the above studies confirmed, in animal models or in healthy volunteer humans, an increase in *Bifidobacteria* and in other beneficial microbes, leading to an increase in total SCFAs [206,207,208]. Other studies, using different probiotics species to enrich the beneficial bacteria count as well as the SCFA amount, obtained the same results in different disease settings, such as colorectal cancer, obesity, T2D, respiratory tract, and cardiovascular diseases [201,205,209,210].

### 7.4. Fecal Microbiota Transplantation

Fecal microbiota transplantation (FMT) is the transfer of feces from a healthy donor into the gut of a recipient to cure a disease associated with gut microbiome imbalance. FMT is a well-established therapy for recurrent *Clostridioides difficile* infection [211] and its complications [212], and has been investigated in other conditions with promising results [213,214].

Several recent lines of evidence suggest that that the increase in SCFAs may be a key determinant of FMT success in different diseases. First, in a mouse model of ischemic stroke, mice transplanted with feces rich in SCFAs, mainly butyrate, experienced an amelioration in neurological symptoms [215].

Moreover, in a randomized controlled trial where FMT from donors with balanced microbiome was more effective than placebo in reducing IBS-related symptoms, FMT increased the fecal SCFA levels, and the post-FMT increase in butyrate levels correlated inversely with symptoms [216]. Finally, in a small pilot trial, FMT from mixed lean donors was able to increase the levels of SCFAs-producing bacteria [217].

Overall, these findings suggest that FMT may be a therapeutic pathway to increase SCFAs in the recipient, especially with the use of targeted donors [218]. However, the real advantage of this therapeutic strategy in clinical practice has yet to be confirmed.

## 8. Conclusions

SCFAs, mainly butyrate, acetate and propionate, play several key roles in human health, from the modulation of the immune system to the regulation of metabolic pathways and the restoration of the gut barrier. Several bacteria are able to degrade substrate sources (mainly non-digestible polysaccharides, but also, less frequently, proteins) to produce SCFAs. The production of specific SCFAs changes over the lifetime, depending on the variations in our diet and consequent shifts in our gut microbiome. SCFAs have been investigated as a therapeutic option in several disorders, with promising findings in pre-clinical models but without satisfactory findings in humans due to the poor colonic availability of oral SCFAs. Moreover, the increase in SCFAs appears to be one of the key therapeutic pathways in several approaches aiming to modulate the gut microbiome, including diet, prebiotics, probiotics, or FMT. Further research, aiming to increase our knowledge of the role of SCFAs in human disorders, investigate new delivery formulations for SCFAs, and disentangle the value of SCFAs as a therapeutic pathway of microbiome modulators, are advocated.

## Figures and Tables

**Table 1:** The role of short-chain fatty acids in different disorders.

Disease	SCFA	Model	Function	Ref.
Inflammatory bowel disease	Acetate	Gpr43−/−, Gpr109a−/−, Nlrp3−/− and Nlrp6−/− mice	Induces NLRP3 inflammosome activation to maintain tissue homeostasis	[133]
	Butyrate	Niacr1+/− Apc min/+ and Niacr1−/− Apc min/+ mice	Increases colonic DCs and macrophages’ production of IL-10, inducing Treg generation	[127]
		Foxp3 ΔCNS1, Foxp3 GFP, Foxp3 Thy1.1 and Gpr109a−/− mice	Promotes Treg differentiation through enhancing Foxp3 activity	[128]
		GPR109a−/− and WT mice	Inhibits AKT and NF-κB p65 signaling pathways in macrophages	[131]
		BMDM cells, C57BL/6 and CX3CR1-GFP/+ mice	Reduces NO, IL-6 and IL-12p40 secretion by macrophages	[132]
		GPR43−/−, Prdm1−/− and WT mice	Increases AREG expression levels in DCs to promote tissue repair	[134]
		Cdx2-IEC monolayer	Induces production of claudin-1 to enhance barrier functions	[138]
	Propionate	Gpr43−/− and Gpr43+/+ mice	Promotes Treg differentiation through GPR-43	[129]
	All SCFAs	HeLa and HEK293 cell lines	Inhibit NF-κB activity through GPR43—βarrestin interactions	[130]
		Isolated human neutrophils, monocytes and PBMC	Promotes anti-inflammatory effects via the regulation of PGE2, cytokine and chemokine release	[58]
		CD4+ T cells and ILCs	Induces production of IL-22 to promote barrier functions	[135]
Colorectal cancer	Butyrate	Caco-2 cell line	Enhances cancer cells’ apoptosis by alterations in the redox state and D-glucose metabolism	[140]
		MCF-7 (T5) and MDA MB 231 cell lines	Arrests cancer cells’ proliferation through upregulation of p21	[141]
	Propionate	Caco-2, HCT116, HCT8, HT-29, SW620, SW480, CBS, FET and MOSER cell lines	Arrests cancer cells’ proliferation through p21 upregulation and decrease in cyclin D3, CDK-1 and CDK-2	[142]
Hypertension	Acetate and propionate	Olfr78−/− and Gpr41−/− mice	Increase blood pressure through Olfr-78	[144]
		Gpr41−/− and WT mice	Reduces blood pressure by binding GRP-41	[145]
	Butyrate	Vagotomized Sheffield strain male Wistar rats	Reduces blood pressure through the regulation of afferent vagal terminals	[146]
Atherosclerosis	Butyrate	ApoE −/− mice	Reduces CCL-2, VCAM-1, and MMP-2 production to stabilize atherosclerotic plaques	[148]
Obesity	Acetate	C57BL/6 male mice	Decreases appetite through central hypothalamic mechanisms	[43]
	Propionate	Isolated human colonic cells	Reduces food intake through the secretion of PPY and GLP-1 via GPR-41	[153]
	Propionate and butyrate	NCI-h716 and HuTu-80 cells	Reduce food intake through the secretion of PPY via inhibition of HDACs	[154]
Metabolic syndrome	Acetate	Isolated adipocytes from GPR43 knockout mice	Decreases lipid plasma levels through inhibition of lipolysis via GPR-43	[159]
	Propionate	Human subjects and in vitro isolated human islets	Enhances glucose-stimulated insulin release and increases β-cell mass	[155]
		Human adipose tissue culture	Decreases lipid plasma levels by stimulating lipogenesis	[160]
	Propionate and butyrate	Stromal vascular fraction of the porcine subcutaneous fat	Stimulates adipocyte differentiation	[156]
	All SCFAs	PPARγ f/f and PPARγ lox/lox mice	Regulate gluconeogenesis and lipogenesis through PPARγ downregulation	[46]
		Male Golden hamsters	Decrease cholesterol plasma levels by enhancing its hepatic uptake	[161]

## Data Availability

Not applicable.
